# Lithiophilic montmorillonite serves as lithium ion reservoir to facilitate uniform lithium deposition

**DOI:** 10.1038/s41467-019-12952-6

**Published:** 2019-10-31

**Authors:** Wei Chen, Yin Hu, Weiqiang Lv, Tianyu Lei, Xianfu Wang, Zhenghan Li, Miao Zhang, Jianwen Huang, Xinchuan Du, Yichao Yan, Weidong He, Chen Liu, Min Liao, Wanli Zhang, Jie Xiong, Chenglin Yan

**Affiliations:** 1 State Key Laboratory of Electronic Thin Films and Integrated Devices, University of Electronic Science and Technology of China, Chengdu, 610054 China; 2 College of Energy, Jiangsu Provincial Key Laboratory for Advanced Carbon Materials and Wearable Energy Technologies, Soochow University, Suzhou, 215006 China; 3 School of Physics, University of Electronic Science and Technology of China, Chengdu, 610054 China; 4 National Key Laboratory of Science and Technology on Advanced Composites in Special Environments Center for Composite Materials and Structures Harbin Institute of Technology, Harbin, 150080 China; 5 Hunan Provincial Key Laboratory of Thin Film Materials and Devices, School of Materials Science and Engineering, Xiangtan University, Xiangtan, 411105 China

**Keywords:** Batteries, Batteries, Batteries

## Abstract

The growing demand for lithium batteries with higher energy densities requires new electrode chemistries. Lithium metal is a promising candidate as the anode material due to its high theoretical specific capacity, negative electrochemical potential and favorable density. However, during cycling, low and uneven lithium ion concentration on the surface of anode usually results in uncontrolled dendrite growth, especially at high current densities. Here we tackle this issue by using lithiophilic montmorillonite as an additive in the ether-based electrolyte to regulate the lithium ion concentration on the anode surface and thus facilitate the uniform lithium deposition. The lithiophilic montmorillonite demonstrates a pumping feature that improves the self-concentrating kinetics of the lithium ion and thus accelerates the lithium ion transfer at the deposition/electrolyte interface. The signal intensity of TFSI^−^ shows negligible changes via in situ Raman tracking of the ion flux at the electrochemical interface, indicating homogeneous ion distribution, which can lead to a stable and uniform lithium deposition on the anode surface. Our study indicates that the interfacial engineering induced by the lithiophilic montmorillonite could be a promising strategy to optimize the lithium deposition for next-generation lithium metal batteries.

## Abstract (web summary)

The address one of the major challenges facing the lithium metal anode, here the authors use lithiophilic montmorillonite as an additive to the ether-based electrolyte to regulate the lithium ion concentration on the anode surface, facilitating uniform lithium deposition.

## Introduction

Lithium metal is the ultimate anode of choice and could push lithium ion batteries to the next performance level^1–4^. Once paired with the high-capacity cathode material (e.g., sulfur^5^, oxygen^6^, etc.), the lithium metal batteries (LMBs) deliver higher specific energy (>500 Wh kg^−1^) at the cell level and a lower cost (below US$100 kWh^−1^) than the traditional lithium ion batteries^7,8^. The practical application, however, is hindered by Li plating on the surface of the current collector (e.g., Cu) since liquid electrolyte is generally adopted, where the concentration of the Li ions would drop to zero based on Chazalviel space charge model during cycling^9,10^. Particularly, at high rates, large space charge and electric field will be induced in the vicinity of electrode surface, rendering uneven Li protuberances^11,12^. During the subsequent deposition process, the protuberances are amplified until dendrites are produced^13^. Thus the practical applications of the LMBs will be blocked by the formed dendrite due to the issues including: (1) the risk of piercing the separator until electronically connecting the electrodes, causing the “soft short”^14^; (2) the consumption of electrolyte^15,16^; and (3) the decay of battery capacity^17,18^.

Optimizing the structure of current collectors is a typical way to suppress dendritic spread via concealing Li deposits (or dendrites) within the inner of current collector^19–21^. Although those methods show certain degree of controllability in decreasing the risk of short circuit, the dendrite problem appears once the height of Li plating exceeds the thickness of designed current structures. Electrostatic shielding is the other compensating mechanism to reduce the Li dendritic growth via accommodating the charge distribution^22,23^. Typically, cationic metal salt with special properties is added in the electrolyte to reduce the electric-field intensity on the surface of the current collector caused by uneven three-dimensional (3D) protrusions^13,24^. However, those cations may be seriously reduced due to the low negative electrochemical potential of Li anode. The reduced cation could be involved in the formation of solid electrolyte interphase (SEI), causing the abatement of electrostatic shielding effect. Beside the above strategies, artificial interface modifications (or solid electrolytes) is another approach to ensure the uniform transport of Li ions through the artificial layer^25–27^, resulting in uniform nucleation on the surface of the current collector. However, the artificial interface modifications are challenging as a result of the complicated preparation process and harsh conditions, leading to somewhat divorcing from industrial applications. Moreover, the material selection for coating on Li anode needs to be carefully designed with insulating nature and higher shear modulus at least twice than that of the metallic Li^3,28^. To this end, effective and feasible pathways to suppress Li dendrites are still urgently required to achieve safe Li anode for practical application.

**Fig. 1:**
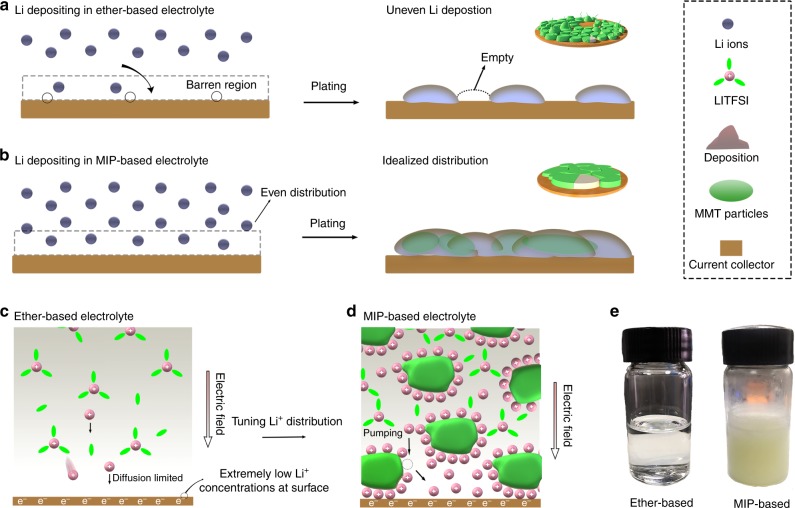
Designing concept of tuning redistribution of Li ion concentration. The nucleation model with barren and rich Li ion concentration at negative surface. Barren Li ion profile in **a** makes the nucleation sites uncontrollable, resulting in uneven Li deposits. Rich Li ion profile distribution in **b** realize idealized nucleation sites. **c** and **d** Schematic illustration describing Li ion plating behavior in ether-based and MIP-based electrolyte, respectively. The diffusion limited of Li ions in ether-based electrolyte results extremely low Li ion concentrations at Cu surface under an electric field. Electro-kinetic pumping phenomena in MIP-based electrolyte allows Li ion self-concentrate at the interface of electrode/electrolyte. The arrows in **c**, **d** represent the movement of the cations. **e** Optical images of ether- and MIP-based electrolyte

Concerning liquid electrolyte (e.g., ether-based electrolyte), the inhomogeneous ion distribution is an important cause of the uneven Li deposition and thus the generation of Li dendrite in liquid electrolyte^10^. As shown in Fig. 1a, the lithium ions in the electrolyte deposit onto the surface of the copper current collector during electroplating. However, due to the influence of the lithium ion migration rate in the electrolyte, ions near the electrochemical interface form a double layer^29^. After reduction on the surface of the electrode, the Li ions in the electrolyte cannot replenish the ions at the interface immediately, resulting in the formation of the low concentration regions (Fig. 1a)^30^. Subsequently, Li will selectively deposit in these regions, causing the uncontrollable lithium accumulation. On the contrary, uniform ion distribution (Fig. 1b) can effectively avoid this situation, leading to uniform deposition of the Li ions based on diffusion model that the electrodeposits display a smooth morphology if ionic concentration profile at the anode evolves to a steady state and high concentration^9,10^.

Montmorillonite, as a member of phyllosilicate, is a classical inorganic material for eliminating heavy metal ion pollution due to the ion self-concentrate that induced higher ionic concentration on its surface rather than that in the bulk solution^31,32^, benefited from its large specific surface area, high ion exchange capability and good adsorption performance. Inspirited by this, herein, by introducing the montmorillonite into the ether-based electrolyte, the lithium ion distribution in the electrolyte can be optimized because of the ionic self-concentrate on the montmorillonite (Fig. 1d), which enable stable and uniform Li deposition on the Cu surface, yielding dendrite-free Li deposits. Compared with the artificial interface modification, the proposed ionic self-concentrating electrolyte is easy to operate, compatible with the mass production, and has the potential to combine with other devices by just replacing the common ether-based electrolyte with the proposed electrolyte without embellishing the lithium anode. Systematic in situ Raman/finite element simulation/other studies reveal the deposition mechanism, providing guidance on the optimization of Li deposition for next-generation LMBs.

## Results

### Design of the ion self-concentration electrolyte

The montmorillonite material is single phase according to X-ray powder diffraction (XRD) analysis. The characteristic diffraction peak at 6.12° (Supplementary Fig. 1) corresponds to an interlayer distance of 1.4 nm, which provides sufficient space within montmorillonite for Li-ion diffusion and electrolyte wetting^25^. After addition of montmorillonite particles, the ether-based electrolyte gradually changes from gray to transparent, implying that the particles cannot be uniformly dispersed in the electrolyte. To ensure uniform distribution of montmorillonite particles in the electrolyte, ionic conducting polymer polyethylene oxide (PEO) was introduced as the framework due to the high binding energy between montmorillonite and PEO (Supplementary Fig. 2). Hereafter, this electrolyte with featured montmorillonite in PEO is referred to as MIP.

Figure 1c, d illustrates the designing concept of tuning the distribution of Li ions. The adsorption and self-concentrating ability of Li ions on the surface of montmorillonite particles are confirmed with the zeta potential method (Supplementary Fig. 3) via which the adsorbing ion-induced surface potential can be detected. The results show that the montmorillonite possesses a strong affinity for Li ions with a high zeta potential (+26 mV, the tested electrolyte is based on 20 mM lithium bis(trifluoromethanesulfonyl)imide (LiTFSI) in 1, 3-dioxolane (DOL)) which can adjust the Li distribution through self-concentrate on montmorillonite surface^2,33^, as shown in Fig. 1d. The ion redistribution profile generates a favorable initial interfacial concentration, C_0_, compared to the pure ether electrolyte (Fig. 1c), effectively delaying Sand’s time to suppress initial uneven Li nucleation under electric field, which is beneficial for forming a stable Li nucleation. Moreover, no deposit is observed in the bottom of the bottle even after resting for ten days, indicating the dispersion stability of the montmorillonite in the electrolyte (Fig. 1e and Supplementary Fig. 4). The structure stability of the montmorillonite was also confirmed by XRD after being soaked it in electrolyte for 24 h (Supplementary Fig. 5).

### Morphology and evolution of Li deposition on Cu negative surface

**Fig. 2:**
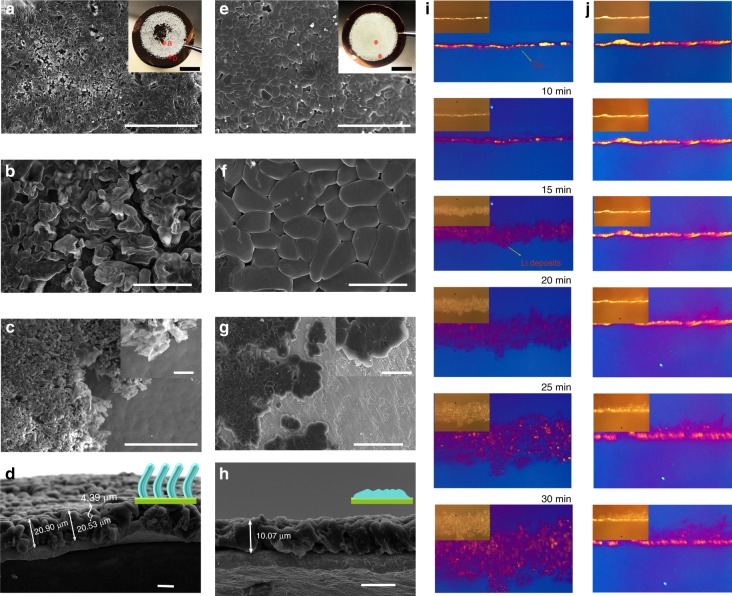
The morphology of Li deposition on Cu surface. SEM images of **a**–**d** and **e**–**h** show the Li deposits in ether-based and MIP-based electrolyte with 2 mAh cm^−2^ at 0.5 mA cm^−2^, respectively. The inset digital images in **a** and **e** show the typical Li deposit distribution after plating without and with tuning Li ion distribution. The SEM images of **a**, **b** and **e**, **f** show ether-based and MIP-based in plane Li deposit region, respectively, marked red a in digital image. **c**, **g** The edge Li deposition (marked red b) of ether-based and MIP-based electrolyte, respectively. **d**, **h** The cross section of Li deposition in ether−based and MIP-based, respectively. The Li deposition in ether-based electrolyte shows columnar structures and the thickness is ~20 μm; The Li deposition in MIP-based electrolyte shows the thickness (~10 μm) is only half of that in ether-based electrolyte, implying more dense lithium formation. the inset image in **d**, **h** shows growth model. **i**, **j** Optical microscope images of the Li deposits in ether (left column) and MIP (right column) -based electrolyte at 0, 10, 15, 20, 25, and 30 min at a current rate of 3 mA cm^−2^. Scale bar, (**a**, **c**, **e**, **g**) 50 μm, (**b**, **d**, **f**, **h**) 10 μm. The scale bar in the inset, (**a**, **e**) 0.5 cm, (**c**, **g**) 10 μm

The plating morphologies of Li deposition using ether electrolytes with and without MIP are captured to investigate the deposition behavior of Li ions (Fig. 2a–h). The surface of Cu foil was first washed with de-ionized water and ethanol to remove the possible impurities. Before Li platting, the Cu surface remains rough and uneven (Supplementary Fig. 6). As shown in Fig. 2a (the inset image), rough surface with a high density of cracks observed in ether-based electrolyte after Li plating with a capacity of 2 mAh cm^−2^ on Cu foil. Furthermore, we select two representative regions at the inner (marked red a) and edge (marked red b) of Li deposits to observe the morphology after Li deposition. For the ether-based electrolyte, porous structure with irregular massif-like 3D Li dendrites can be clearly found (Fig. 2b). Even worse, the Li deposits show sharper corners and present seaweed morphology at the edge region (Fig. 2c). It is easier for residual on separator of such sharp structures of Li dendrites at the edge region to form “dead Li” (Supplementary Fig. 7). The thickness of deposits reaches up to 20 μm with a needle-like structure (Fig. 2d). In contrast, the Li deposits on the Cu collector in the MIP electrolyte display uniform surface without sharp structure even at the edge regions (Fig. 2g), and the thickness is only 10 μm with a highly dense distribution (Fig. 2h), clearly demonstrating the homogeneous Li plating is associated with the ionic self-concentrating on the montmorillonite. The element mapping displays the montmorillonite is involved in the Li deposition (Supplementary Fig. 8). Therefore, scanning electron microscope coupling with focused ion beam (FIB-SEM) was employed to analysis the Li deposits (Supplementary Fig. 9). It can be clearly observed that the montmorillonite can be served as heterogeneous nucleation seeds for adsorbing Li ions to enable a uniform deposition rather than forming large cluster or Li dendrites due to the low diffusion energy barrier for Li ion migratory.

Additionally, a Li||Cu cuvette-type optical cell was also fabricated to investigate the morphology of the Cu surface in both electrolytes during the Li deposition process through in situ optical microscopy. The images recorded at different stages are displayed in Fig. 2i, j. The Li||Cu cell was subjected to a high current density of 3 mA cm^−2^. In the beginning, the surface of Cu is smooth and bright in the ether-based electrolyte. However, Li dendrites appear immediately upon application of the current. As time goes on, numerous moss-like dendrites are formed on the Cu surface. In comparison, uniform electrodeposition of Li is obtained in the MIP-based system at high current density, leading to a flat surface without dendritic structures. This observation suggests that the MIP-based electrolyte can effectively suppress the formation of Li dendrites in a Li-metal rechargeable battery.

### Li plating/stripping performance on Cu foil in MIP electrolyte

**Fig. 3:**
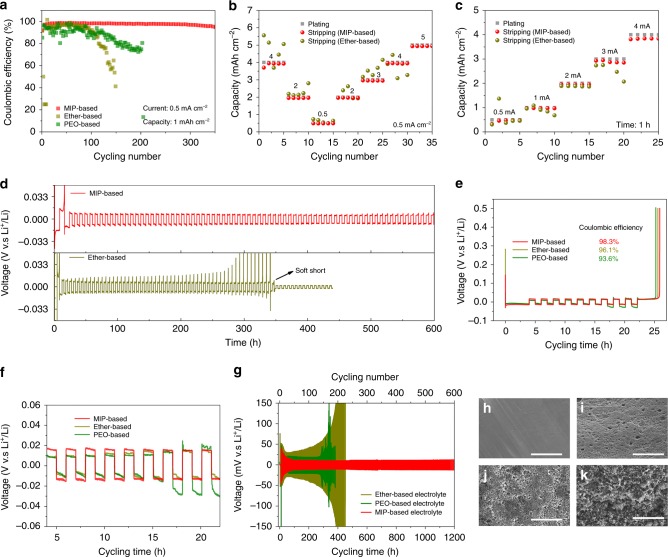
Electrochemical cycling performance of Li||Cu and Li||Li cells. **a** Cycling performance of Li||Cu with 1 mAh cm^−2^ at current density of 0.5 mA cm^−2^, and **b** the corresponding rate performance. **c** The rate performance with different current density at one hour. **d** The symmetrical tests of Li||Cu where 4 mAh cm^−2^ and 3 mAh cm^−2^ Li were firstly deposited on Cu surface for MIP and ether-based electrolyte, respectively, and then half Li deposits are conducted. **e** Voltage-time curves to calculate the average Coulombic efficiency of Li||Cu cells at 0.5 mA cm^−2^. **f** The enlarged view of **e** from 4 ~ 24 h. **g** Charge–discharge curves of symmetrical Li||Li cells at 1 mA cm^−2^. The morphology of Li anodes after plating in fresh (**h**), MIP-based (**i**), PEO-based (**j**), ether-based (**k**) electrolyte. Scale bar, 50 μm

Li||Cu cells are fabricated to examine the cycling stability and Coulombic efficiencies for different electrolytes. Herein, the capacity of 1 mAh cm^−2^ Li deposits is plated on the surface of Cu foil, and then stripped during the charge to 0.5 V. When operated at 0.5 mA cm^−2^, the Li||Cu cell with the MIP electrolyte demonstrates a stable plating/stripping voltage profile and a steady Coulombic efficiency of 98% for more than 300 cycles (Fig. 3a). In comparison, the cells with ether-based electrolyte display obviously inferior Coulombic efficiencies and very fluid charge/discharge voltage profiles (Supplementary Fig. 10). Furthermore, the benefits of MIP-based electrolyte are examined with harsh terms via rate performance in which the same current density (0.5 mA cm^−2^) for different capacity (0.5–5 mAh cm^−2^) and the same platting/stripping time (1 h) for different current density (0.5–4 mA cm^−2^). Clearly, the MIP-based electrolyte can maintain stable plating/stripping voltage profiles even at the high platting capacity of 5 mAh cm^−2^ (Fig. 3b) and high platting/stripping current density of 4 mA cm^−2^ (Fig. 3c). Subsequently, 4 mAh cm^−2^ and 2 mAh cm^−2^ Li are deposited on Cu surface for MIP and ether-based electrolyte, respectively, and then 2 mAh cm^−2^ and 1.5 mAh cm^−2^ Li deposition for MIP and ether-based electrolyte, respectively, are conducted for symmetrical tests. As shown in Fig. 3d and Supplementary Fig. 11, the cell with MIP-based electrolyte exhibits a stable voltage plateau more than 600 h. In sharp contrast, the cell using ether-based electrolyte delivers severe voltage hysteresis as the cycling time is only 250 h (Fig. 3d). The severely fluctuation of voltage profiles may generate from the reiterated formation/extinction of lithium dendrites, resulting in the electrical disconnection.

The average Coulombic efficiency of Li||Cu cell is investigated according to the way reported by Aurbach et al.^34^. As shown in Fig. 3e, the cell with MIP-based electrolyte delivers average Coulombic efficiency of 98.6%, which is much higher than that in both the ether-based (96.1%) and PEO-based (93.6%) electrolyte. The higher Coulombic efficiency of the cell with MIP-based electrolyte reveals the lithium utilization can be improved during Li deposition/dissolution. Additional electrochemical impedance (EIS) spectroscopy was conducted for the Li||Cu cells after plating lithium with a capacity of 1.0 mAh cm^−2^ at a current density of 1.0 mA cm^−2^ (Supplementary Fig. 12). After cycling, the cells with MIP-based electrolyte exhibited a small charge transfer impedance (R_ct_), while the impedances of the cell with ether-based electrolyte fluctuated largely, thus demonstrating the importance of the stable interfacial impedance induced by the MIP-based electrolyte.

The symmetrical Li||Li cells are also employed for exploring the long-standing operation stability during Li plating/stripping. As displayed in Fig. 3g, the cell with MIP-based electrolyte holds a stable cycling for 1200 h at a current density of 1.0 mA cm^−2^, presenting a steady Li plating and striping with a slight polarization (10 mV). However, the cells with other electrolyte exhibit severely fluctuating voltages, which can be attributed to the ever-changing and repeated growth/corrosion interfaces between Li anode and electrolytes (Supplementary Fig. 13). The large voltage hysteresis at early stage mainly originates from the nucleation of lithium deposition and the formation of initial SEI to passivate the lithium anode^35,36^. Figure 3h–k shows the morphology of Li anodes after plating in MIP-, PEO- and ether-based electrolyte, respectively. Clearly, smooth and flat morphology can be observed on both the surface and cross-sectional areas of the deposited Li in the MIP-based electrolyte (Supplementary Fig. 14). Moreover, the symmetrical Li||Li cells still deliver stable Li plating/stripping in the MIP-based electrolyte for 200 h at 1.0 mA cm^−2^ after resting MIP-based electrolyte for one month in glove box, confirming the stability of the montmorillonite dispersed in the electrolyte (Supplementary Fig. 15). Lithium-sulfur battery with MIP-based electrolyte demonstrates a higher average Coulombic efficiency (99.62%) than that of the battery using ether-based electrolyte (95.63%) during 100 cycles, further revealing the role of montmorillonite in the electrolyte to stabilize Li metal anode (Supplementary Fig. 16).

### Transfer mechanism of lithium ions in MIP-based electrolyte

**Fig. 4:**
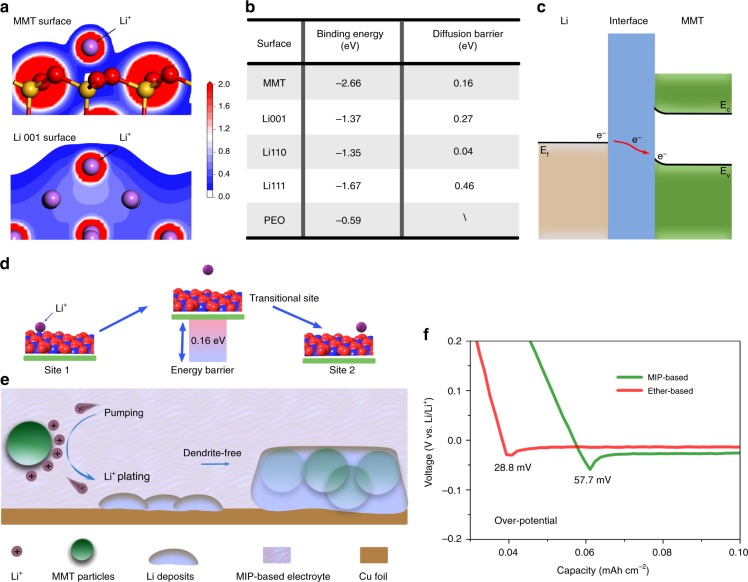
Lithium ion transfer mechanism on montmorillonite surface. **a** The charge distribution of Li ions on montmorillonite and Li <001> surface, respectively. **b** The binding energy and diffusion barrier of Li ion on different material surface. **c** The schematic energy band diagram at the Li/montmorillonite hetero-junction upon contact that shows electron transfer at the interface. **d**, **e** Migrating behavior of Li ions on montmorillonite surface and bulk electrolyte, respectively. **f** The over-potential of Li||Cu cell using MIP- and ether-based electrolyte

To better understand the Li ion transfer behavior, density functional theory (DFT) calculations are carried out. In Fig. 4a, the charge density distribution of Li ions adsorbing on the montmorillonite and Li <001> surface was investigated, showing that the interaction between montmorillonite and lithium ions is of ionic nature, while the bond between Li ion and lithium is metallic. Subsequently, the binding energies for different surfaces are compared. The binding energies of Li ions on montmorillonite surface, Li <001>, Li <110>, Li <111> surface and PEO are 2.66, 1.37, 1.35, 1.67, and 0.59 eV, respectively (Fig. 4b). The higher binding energy of Li ions on montmorillonite than that on lithium crystal plane suggests that the Li ions are adsorbed preferentially on the surface of montmorillonite rather than onto the Li surface during Li deposition. Once adsorption, the Li ions tend to accumulate to form large dendrites, or spread out uniformly with no dendrite generation, depending on the diffusion property of the Li ions on the adsorbents. To further investigate the Li ion diffusion behavior, the migratory barriers of Li ions on various surfaces are calculated and compared. The calculated results indicate the Li ions preferred aggregate on the Li <111> crystal plane due to the highest diffusion barriers compared with Li <001>, Li <110> crystal plane (Supplementary Fig. 17), causing higher concentration at its tip, consistent with the reported value^37^. In contrast, the montmorillonite owns unique layered structure with low migratory barrier (0.16 eV), indicating that the adsorbed Li ions on surface of montmorillonite can easily diffuse and distribute uniformly (Fig. 4d). The energy band diagram demonstrates that once Li and montmorillonite contact with each other, the montmorillonite can serve as heterogeneous nucleation seeds for wetting lithium metal due to the electron transfer at the interface^38^, leading to dendrite-free morphology (Fig.4c). Overall, transfer mechanism of lithium ions in MIP-based electrolyte and the following deposition on the Cu surface can be depicted in three steps (Fig. 4e): (1) Because of the stronger affinity of montmorillonite (2.66 eV) than both metallic Li and PEO, Li ions will self-concentrate near the surface of montmorillonite, in accordance with the trend in the Zeta potential (+26 mV). (2) The adsorbed Li ions will detach, resulting in slightly elevated over-potential (~57.7 mV) as shown in Fig. 4f, then migrate from montmorillonite to the surface of the Cu current collector to produce Li deposits. (3) Because the montmorillonite is immobilized by PEO in the electrolyte, the formed Li deposits can contact and wet the surface of montmorillonite particles (Supplementary Figs. 7–8), and then grow along the surface, resulting in dendrite-free anodes.

### Finite element simulation (FES) of Li-ion concentration profiles and current density vector (CDV)

**Fig. 5:**
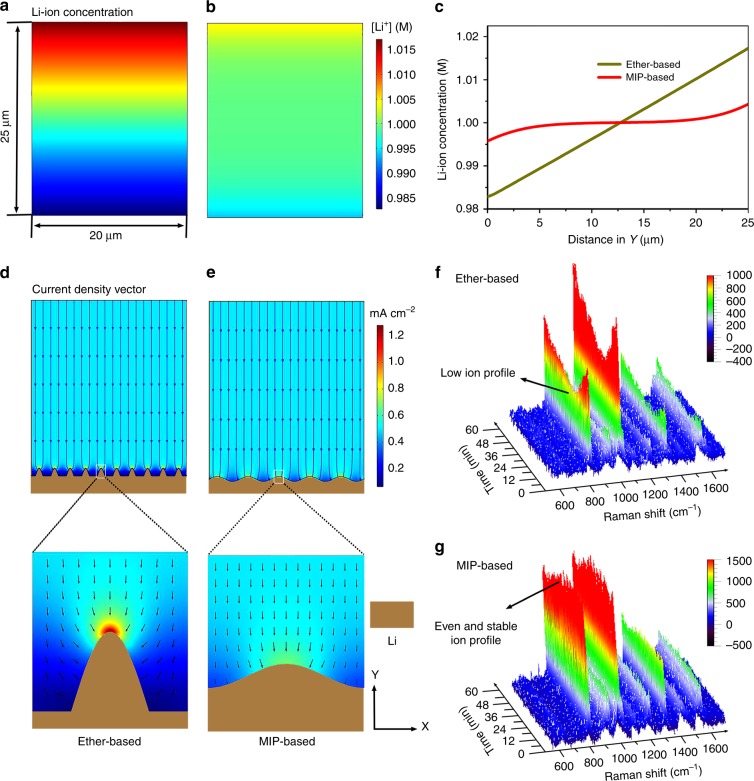
Finite element simulation and in situ Raman to observe the interface information. **a**, **b** Equilibrium Li-ion concentration profiles with constant-reaction-current electrode surfaces at the 20 × 25 µm^2^ scale for ether-based (**a**) and MIP-based (**b**) electrolyte. **c** Li-ion concentration along the Y direction in (**a**, **b**). **d**, **e** Current density vector profiles with constant current 0.5 mA cm^−2^ at the 20 × 25 µm^2^ half cell electrodeposition system for ether-based (**d**) and MIP-based (**e**) electrolyte. The X and Y axes represent the directions parallel and perpendicular to the electrode, respectively. **f**, **g** In situ Raman spectra of ether and MIP-based electrolyte, respectively. The Raman band at 720–760 cm^−1^ is ascribed to the TFSI^−^ band

The FES is then used to study the kinetic equilibrium of Li-ion concentration and the CDV with a constant Li consumption. As shown in Fig. 5a, the lithium ion distribution within the ether-based electrolyte exhibits a typical concentration polarization region with a thickness of ~5 μm along the y axis, where the lithium ion concentration is significantly lower than its initial concentration C_0_ (1 M). The lower lithium ion distributions at the interface of the current collector/electrolyte than that at other regions are more likely to result in the selective deposition of lithium ions, leading to the growth of dendrite during Li platting^9^. In contrast to the ether-based electrolyte, the concentration polarization of lithium ions within MIP-based electrolyte are significantly compressed (Fig. 5b) due to the self-concentrate of lithium ions on the surface of montmorillonite which pumps lithium ions during plating. As a result, the lithium ion concentration at the current collector/electrolyte interface in MIP-based electrolyte is enriched causing homogeneous distribution of lithium ion at the surface of anode. Figure 5c shows the lithium ion concentration along the y direction of the electrode surface in both the ether- and MIP-based electrode. Clearly, the lithium ion concentration is more stable in the MIP-based electrolyte than that in the ether-based electrolyte, suggesting the uniform lithium ion distribution within the MIP-based electrolyte due to its self-concentrate on the montmorillonite surface, which will bring even Li plating and thus dendrite-free Li anode.

The temporal evolution of Li plating is subsequently performed via studying the CDV. The simulated structural model of Li deposition (Fig. 5d, e and Supplementary Fig. 18) are designed based on the experimentally observed morphology of the as-deposited Li with needle-like and flat surface (Fig. 2d, h) in ether- and MIP-based electrolyte, respectively. Profiling of the Li-ion concentration (Supplementary Fig. 18) shows the Li-ions in the ether-based electrolyte tend to accumulate at the top of the Li protuberance (Supplementary Fig. 18a), causing unevenness of ion distribution (Supplementary Fig. 19). In contrast, in MIP-based electrolyte (Supplementary Fig. 18b), the Li-ions displays more even distribution at the interface, which benefits the Li depositing. By studying the CDV line, it can be found that the current density (>1.2 mA cm^−2^) at the top of the Li protuberance in the ether-based electrolyte (Fig. 5d) far exceeds the simulated constant current density (0.5 mA cm^−2^), and such a high current density will further aggravate the growth of lithium dendrites. Moreover, even at a high current density of 3 mA cm^−2^ (Supplementary Fig. 20), the MIP-based electrolyte still delivers more uniform surface current distribution, enabling a dendrite-free Li anode.

### In situ Raman observation of the electrochemical interface during plating process

The visualization of even Li ion profile distribution in MIP-based electrolyte at the Cu negative interface is further demonstrated via in situ Raman spectroscopy measurement in which the Raman band at 741.8 cm^−1^ is ascribed to the coupled CF_3_ bending and S-N stretching in TFSI^−^ (Supplementary Fig. 21)^39^. For ether-based electrolyte, we observe a significant change of the spectrum intensity with increasing Li plating capacity. At the early stage of plating, the Raman beam mainly focused on the neighborhood of Cu surface (Supplementary Fig. 22a), and the detected signal is ascribed to the bulk electrolyte, resulting in considerable intensity of Raman signal. The signal intensity, however, dramatically decreases with the growth of Li deposits. Especially, the intensity reduces to half of the initial value after plating for 30 min because the interface gradually moves close to the focused point of Raman beam (Supplementary Fig. 22a). The decreased signal intensity at this time presents the real ion distribution at the interface,^29^ providing strong evidence for uneven ion distribution on the anode surface compared with the bulk electrolyte (Supplementary Fig. 22b). In contrast, benefiting from the pumping feature of montmorillonite for self-concentrate of Li ions, ion distribution at the electrochemical interface is not affected, which is confirmed by the nearly constant intensity of Raman signal (Supplementary Fig. 23). The increased ion concentration (Supplementary Fig. 24) favors further delay of the Sand’s time and provides stable and even plating.

## Discussion

In summary, we proposed a promising strategy to tune ion distribution for Li dendrite-free deposition by introducing montmorillonite into the bulk electrolyte as nucleophilic medium. The redistribution of Li ion effectively suppresses the formation of ion barren region on the surface of anode and further delays Sand’s time by increasing the ion concentration at the electrode/electrolyte interface. This mechanism is demonstrated by FES and in situ Raman measurement which shows that the intensity at the deposition/electrolyte interface in ether-based electrolyte is significantly lower than that of the bulk electrolyte at the high current density of 3 mA cm^−2^. In contrast, for MIP-based electrolyte, flat and stable Raman spectra can be observed during Li plating, which means the barren area has been turned into fertile fields by the MIP-based electrolyte. The fertile ion distribution can offset the negative effects induced by inhomogeneity on the surface of the current collector. As the deposition continues, the montmorillonite serves as the heterogeneous nucleation seeds for adsorbing Li ions, leading to a uniform deposition rather than large cluster or Li dendrites. The stable Li platting/stripping renders an enhanced electrochemical performance even at high platting/stripping capacity of 5 mAh cm^−2^, and the Li||Cu cell can also deliver a high Coulombic efficiency. When employing Aurbach’s methods for probing the Coulombic efficiency^34^, the average Coulombic efficiency of the cell with MIP-based electrolyte can reach up to 98.3%, which is much higher than that of the cell using ether-based electrolyte (96.1%). This study suggests that the distribution of Li ion at the deposition/electrolyte interface is a decisive factor for Li ion plating. Guiding even Li ion distribution in bulk electrolyte by nucleophilic inorganic-interlamellar montmorillonite material offers a promising route to suppress dendrite growth and enable ultra-stable Li platting/stripping.

## Methods

### MIP-based electrolyte preparation

In an Ar-filled glove box, 3.3 μM PEO (molecular weight: 300,000 g mol^−1^, Aladdin) was dissolved in 10 mL ether-based electrolyte for which 1.0 M lithium bis(trifluoromethylsulfonyl)imid (LiTFSI) was dissolved in DOL/DME (1:1 by volume; DOL = 1,3−dioxalane; DME = dimethylether) with 1 wt% LiNO_3_, and then 100 mg KFS-type montmorillonite particles (Aladdin) were added. The obtained colloidal solution was subsequently stirred for 24 h at 60 °C to ensure uniform dispersal. After that, the milk white solution, denoted as montmorillonite implant in PEO (MIP)-based electrolyte, was obtained and left in glove box for following electroplating processes and electrochemical long-term cycling tests.

### Materials characterization

The structure of montmorillonite was characterized by powder X-ray diffraction (XRD) at room temperature using a UltimaIV diffractometer with CuKα1 radiation (*λ* = 1.4506 Å) and a position-sensitive detector. Zeta potential was used to measure the shear plane’s potential using NanoBrook 90Plus PALS Particle Size Analyzer (USA). The morphology of Li deposits was observed using a SEM (FEI, NANOSEI 450), operated at 3.0 kV with the SEM samples were prepared in the glove box.

### Calculations methods

The interaction among PEO, montmorillonite, Li^+^, DOL, and DME are calculated by the DFT implemented by DMOL3 software. Double numerical plus polarization (DNP) basis with DFT Semi-core Pseudopots treatment and a GGA-PBE function were employed in the self-consistent field calculation with a SFC tolerance of 1 × 10^−6^. A short polymer chain of C_8_H_2_O_4_ was used to stand for PEO polymer.

### Finite element simulation

The COMSOL Multiphysics software, coupling with lithium battery module simulation, was employed for the FES. The plating morphological evolution was conducted in 20 × 25 μm. The electrolyte conductivity is 10^−2^ S m^−1^, the diffusion coefficient is 1.5 × 10^−10 ^m s^−2^, the lithium ion concentration is 1 M, and the surface current densities of 0.5 and 3 mA cm^−2^ are simulated. For the case of modification, a porous adsorption medium is added to the electrolyte, and the adsorb ability (2.66 eV) of montmorillonite for lithium ions is based on the DFT calculations.

### In situ Raman measurement

Li||Cu cell with a quartz window was used for in situ micro-Raman spectroscopy analysis. The confocal micro-Raman spectrometer was equipped with a 785 nm laser source. Each spectrum was the average of two accumulations at a resolution of approximately 4 cm^−1^. The Raman beam is foucsed on the neiborhood of Cu cross section. To ensure enough Li plating on Cu surface, 3 mAh cm^−2^ Li was plating at a current density of 3 mA cm^−2^. All spectral analyses were carried out using build-in commercial software of micro-Raman spectrometer. All Raman spectra are shown as measured without background correction.

### Electrochemical cycling tests

The Li||Cu cells and symmetrical Li||Li cells were assembled in the argon filled glove box. The coin cells (CR-2032) were used to test the electrochemical performance. The used separator is polypropylene membranes (Celgard 2400). The coin cells were monitored using a battery cycler on CT2001A cell test instrument (Wuhan LAND Electronic Co., Ltd). And the Z−K study was performed using CHI660E (Shanghai Chenhua instrument Co., Ltd) electrochemical workstation. The average Coulombic efficiency of Li platting/stripping was calculated based on Aurbach et al.^34^. Briefly, a capacity of 2 mAh cm^−2^ Li deposition was first platted on the surface of Cu electrode. Then, the Li deposition with 0.5 mAh cm^−2^ was stripped and re-deposited at a current density of 0.5 mA cm^−2^ for 10 cycles. The cell was stopped till the voltage exceeded 0.5 V vs. Li/Li^+^ as the final stripping process. The following equation is employed for calculating the average cycling efficiency.

1 $${X} = \left[ {1 - \frac{{X\left( {Q_1 - Q_r} \right)}}{{Q_cN}}} \right] \times 100$$

where *X* presents the cycling efficiency (%), *N* is the number of cycles, *Q*_1_ is the initial Li deposition (initial loading), *Q*_*r*_ is the finally residual of Li deposition (final charging), *Q*_*c*_ is the charge for the single amount of Li deposition (half cycle). For lithium sulfur battery tests, the preparation of sulfur electrode is followed our previously reported method.^40^ Typically, the sulfur content in S/C composite is 60% and the slurry of S/C and Poly(vinylidene fluoride) binder in a mass ratio of 9:1 in N-methyl pyrrolidone solvent was casted on the surface of the carbon coated aluminum foil. The use of electrolyte is MIP- or ether-based electrolyte.

## Supplementary information

Supplementary Information

## Data Availability

The authors declare that all the data supporting the findings of this study are available within the article and its Supplementary Information or from the corresponding author upon reasonable request.
