# Supplementary material for: Lithiophilic montmorillonite serves as lithium ion reservoir to facilitate uniform lithium deposition
Source: Nat Commun. 2019 Oct 31;10:4973. doi: 10.1038/s41467-019-12952-6 (PMC6823444; doi:10.1038/s41467-019-12952-6)
Supplement: Supplementary file 1 — Supplementary Information [file 41467_2019_12952_MOESM1_ESM.pdf]

Supplementary Information

Lithiophilic montmorillonite serves as lithium ion reservoir to facilitate uniform lithium deposition

Chen et al.

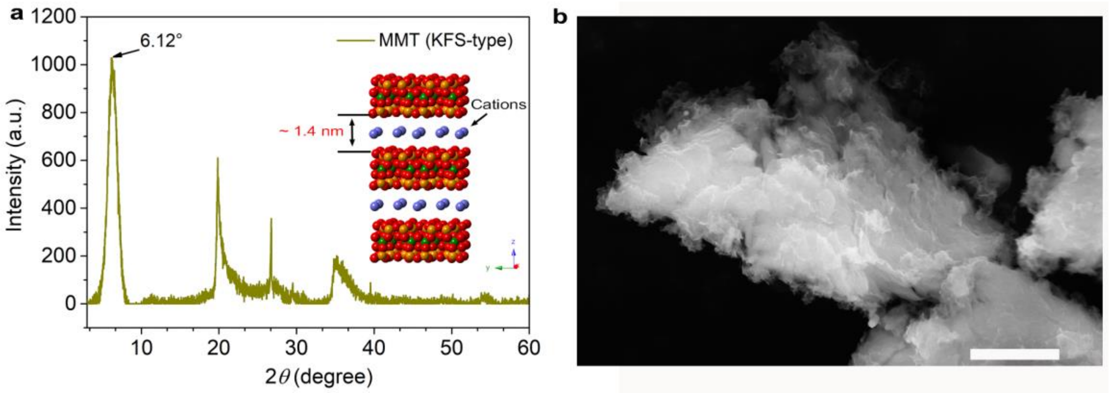

Supplementary Figure 1 | **Structure characterization of montmorillonite materials**. **a** XRD spectrum for KFS-type montmorillonite, and the characteristic peak of interlayer loacted at 6.12° represents the distance of about 1.4 nm that can allow the cations transfer freely, showing in the inset image. **b** SEM image of the montmorillonite materials. Scale bar, 2.5 μm.

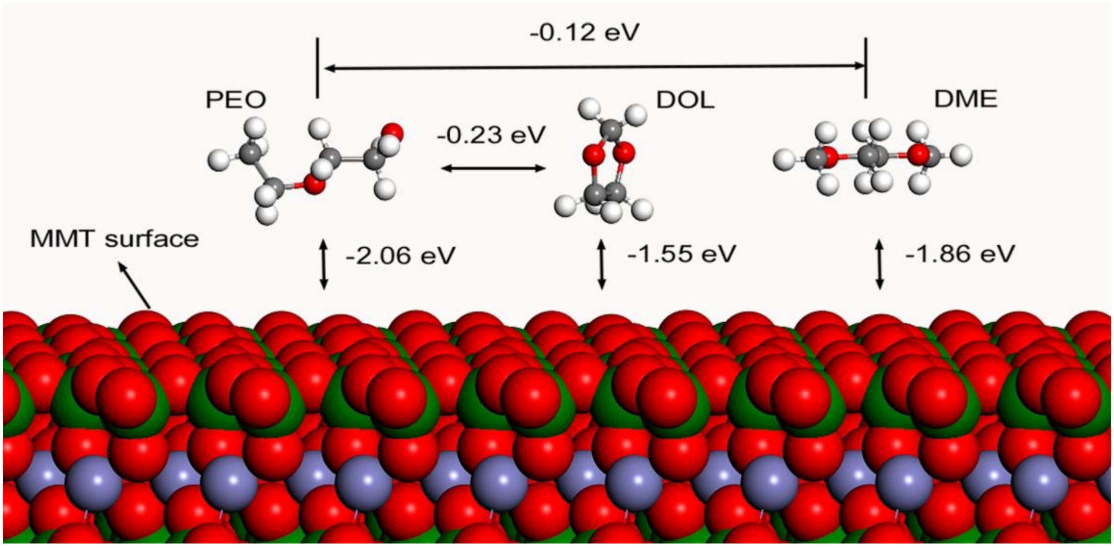

Supplementary Figure 2 | **The DFT caculations for the binder energy between montmorillonite, PEO, DOL, as well as DME, respectively.**

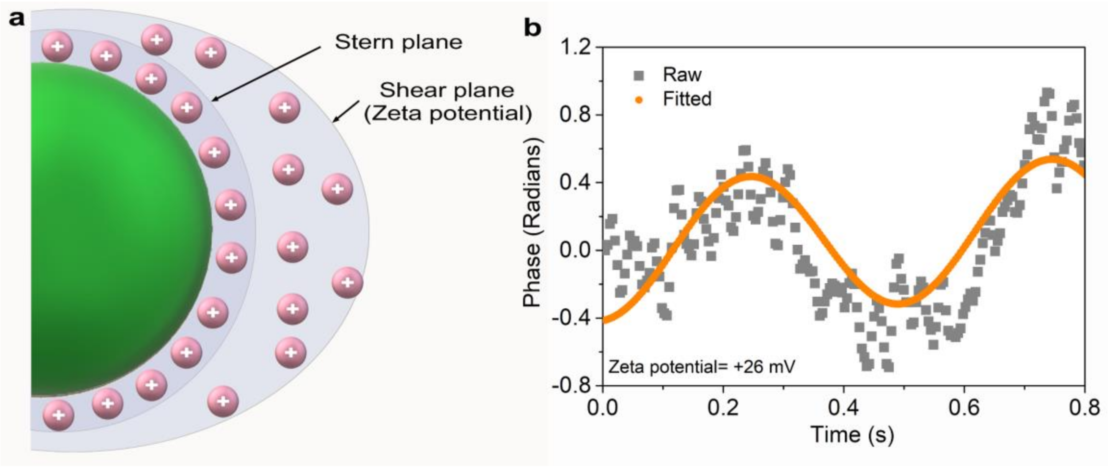

Supplementary Figure 3| **Schematic diagram of high-zeta-potential montmorillonite particles with negative surface charge**. **a** The pink sphere represents the attracted cations. **b** The measured zeta value of montmorillonite particles, which are dispersed in DOL solvent with 20 mM LiTFSI.

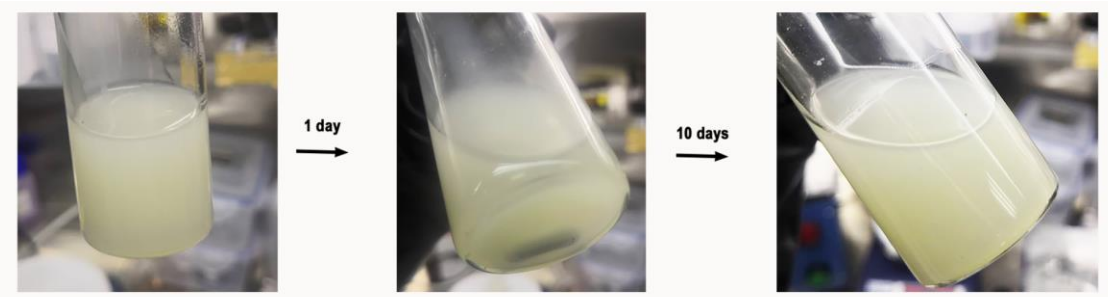

Supplementary Figure 4 |**Optical images of MIP-based electrolyte**. no precipitations were observed even resting for 10 days.

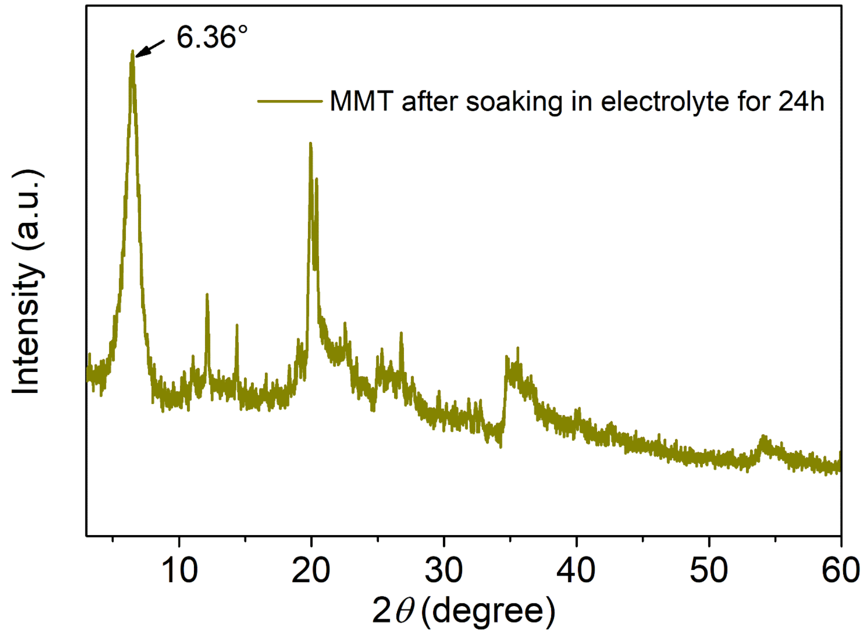

Supplementary Figure 5 | **Structure characterization of montmorillonite materials after soaking in ether-based electroyte for 24 hours.** The result shows that the structure of montmorillonite did not change, confirming the stability of montmorillonite after soaking it in the electrolyte.

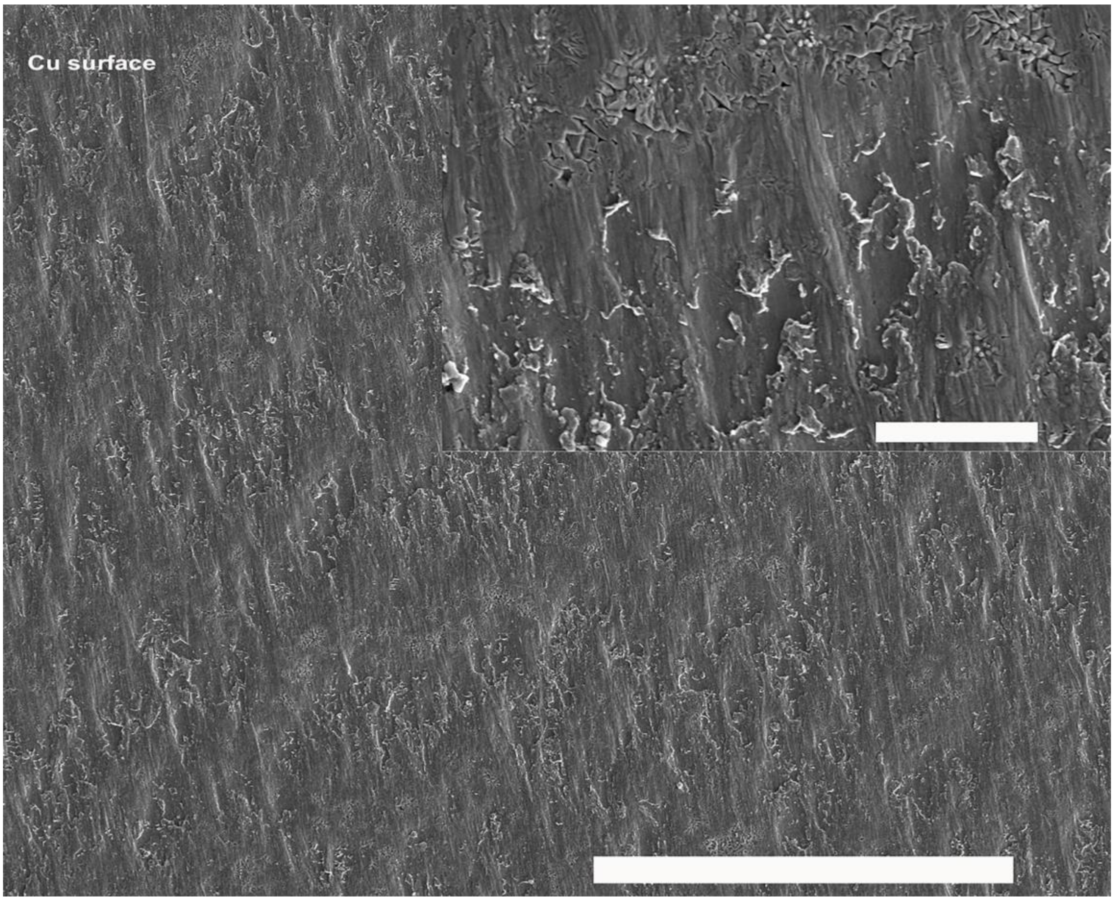

Supplementary Figure 6 | **SEM images for Cu surface.** Scale bar, (low-resolution) 100 μm, (high-resolution) 10 μm.

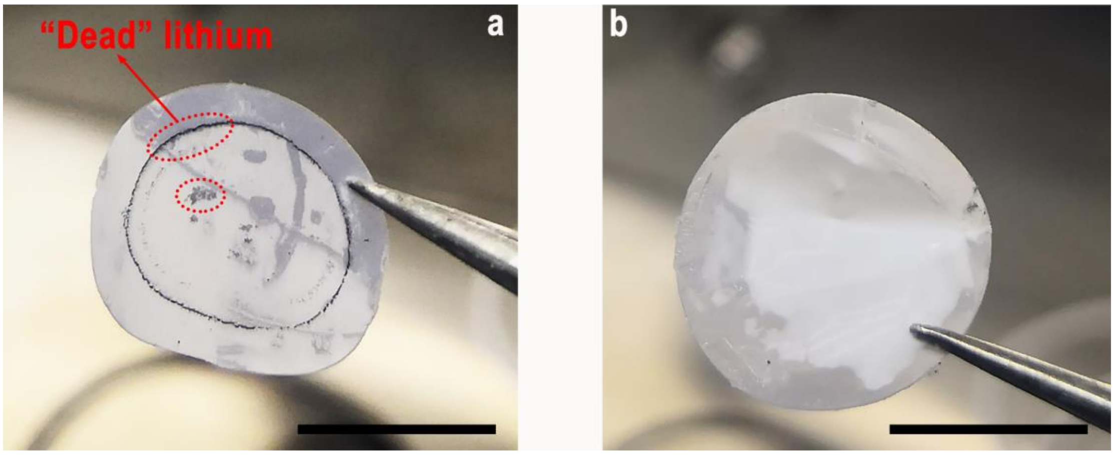

Supplementary Figure 7 | **Optical images of the separator after Li plating.** The electrolyte is based on **a** ether- and **b** MIP-based electrolyte, respectively. "Dead" lithium can be found in ether-based electroylte. Scale bar, 10 mm.

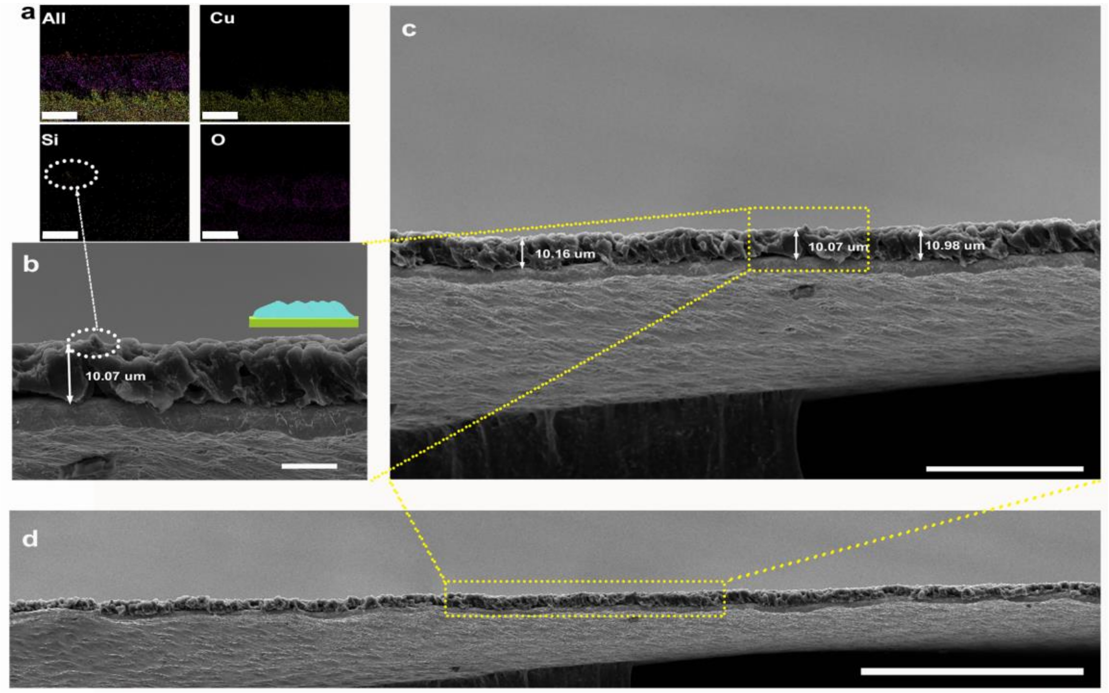

Supplementary Figure 8 | **Section SEM image of the Li deposits on Cu surface in MIP-based electrolyte with a capacity of 2 mAh cm^{-2} at 0.5 mA cm^{-2} and corresponding mapping.** The montmorillonite materials can be found in the deposited Li inner, marked with arrow in **a**. the thickness of 10 μm is found, implying more dense lithium formation in MIP-based electrolyte than that formed in ether-based electrolyte. Scale bar, (**a, b**) 10 μm, (**c**) 50 μm, (**d**) 200 μm.

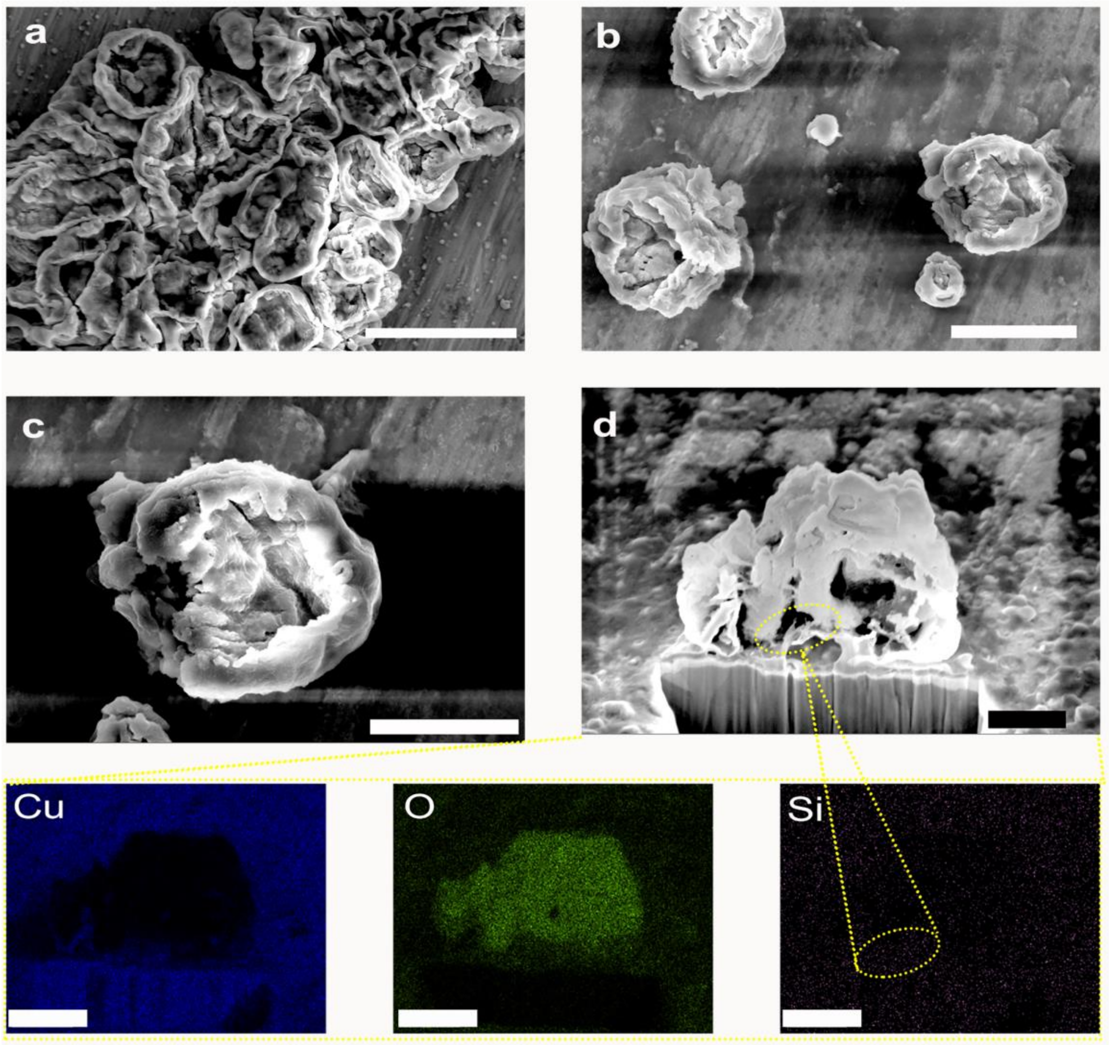

Supplementary Figure 9 | **FIB-SEM analysis of Li deposits using MIP-based electrolyte. a-c** The SEM images for Li deposition with low plating capacity of 0.2 mAh cm^{-2}. **d** The cross-section carved with a FIB. The yellow frame suggests a central region within the cross-section that was selected for further analysis with EDX where the unique silicon signal of montmorillonite can be found. Scale bar, (**a**) 10 µm, (**b**) 5 µm, (**c**) 3 µm, (**d**) 1 µm.

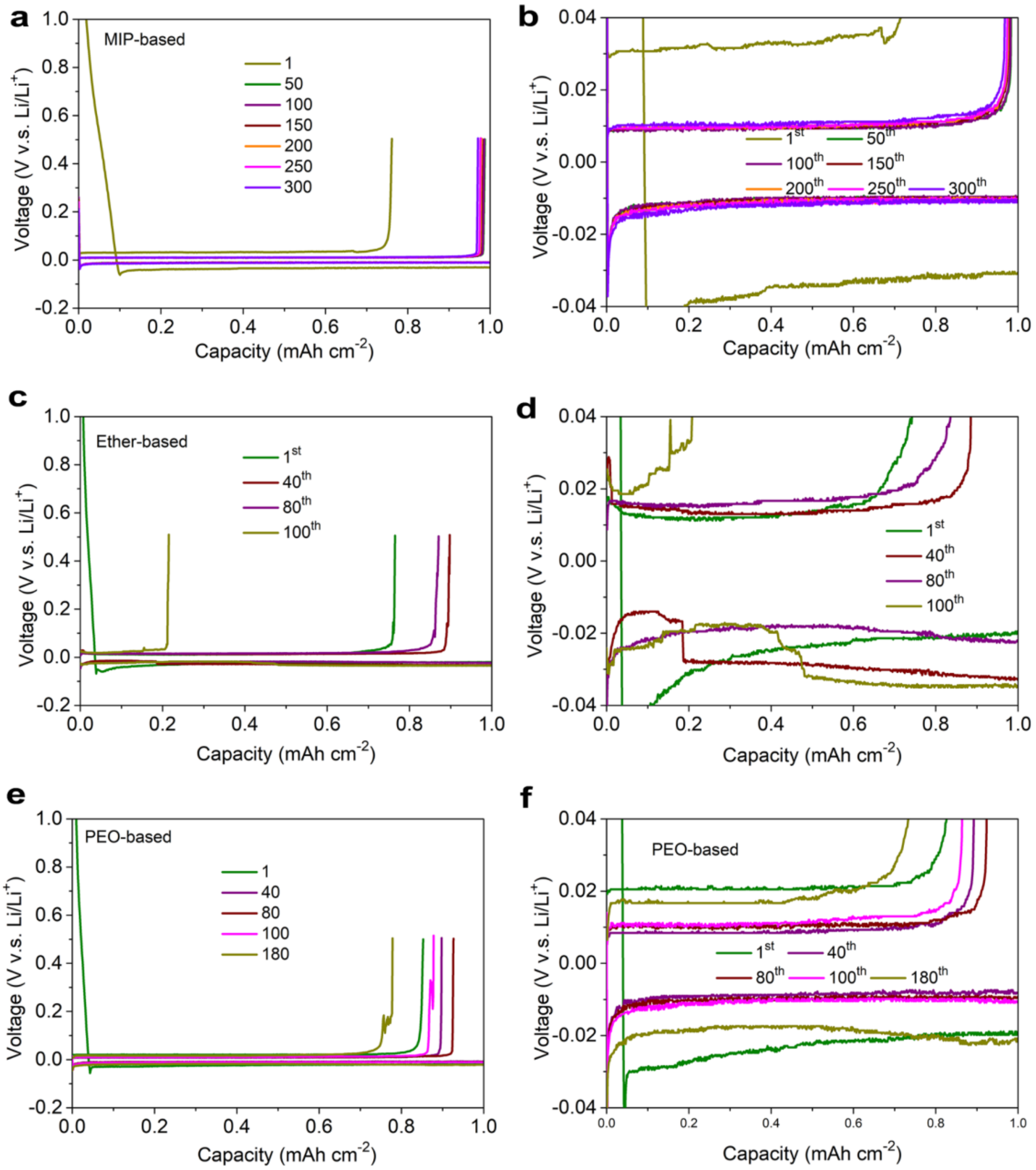

Supplementary Figure 10 | **The voltage plateau of Li||Cu cells with different electrolyte.** (**a, b**) MIP-, (**c, d**) Ether-, (**e, f**) PEO-based eelctrolyte.

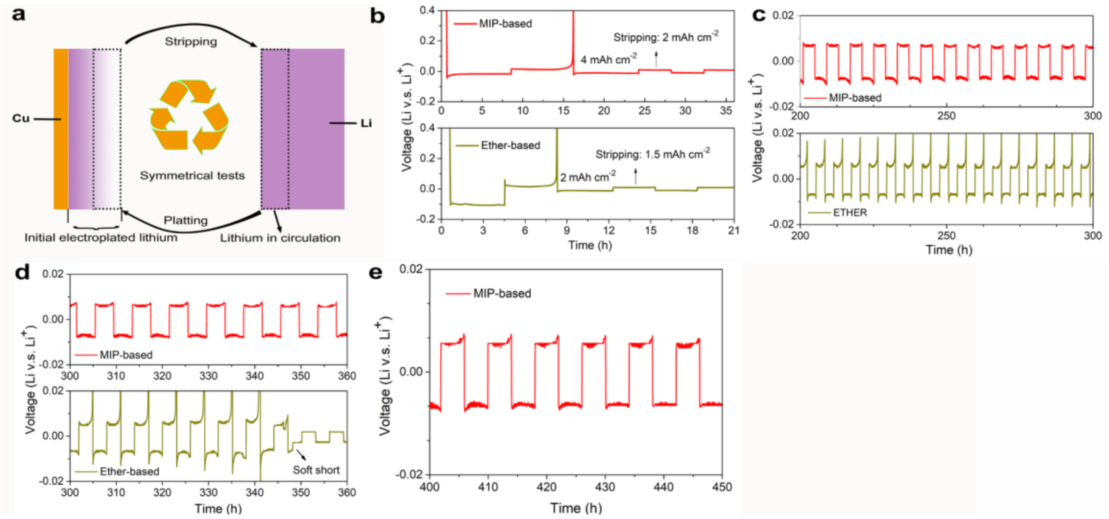

Supplementary Figure 11 | **Li||Cu symmetrical tests. a** The Schematic diagram where 4 mAh cm^{-2} and 2 mAh cm^{-2} Li are firstly deposited on Cu surface for MIP- and ether-based electrolyte, respectively, and then 2 mAh cm^{-2} and 1.5 mAh cm^{-2} Li deposition for MIP- and ether-based electrolyte, respectively, are conducted for symmetrical tests. **b-e** The enlargement for different cycling time.

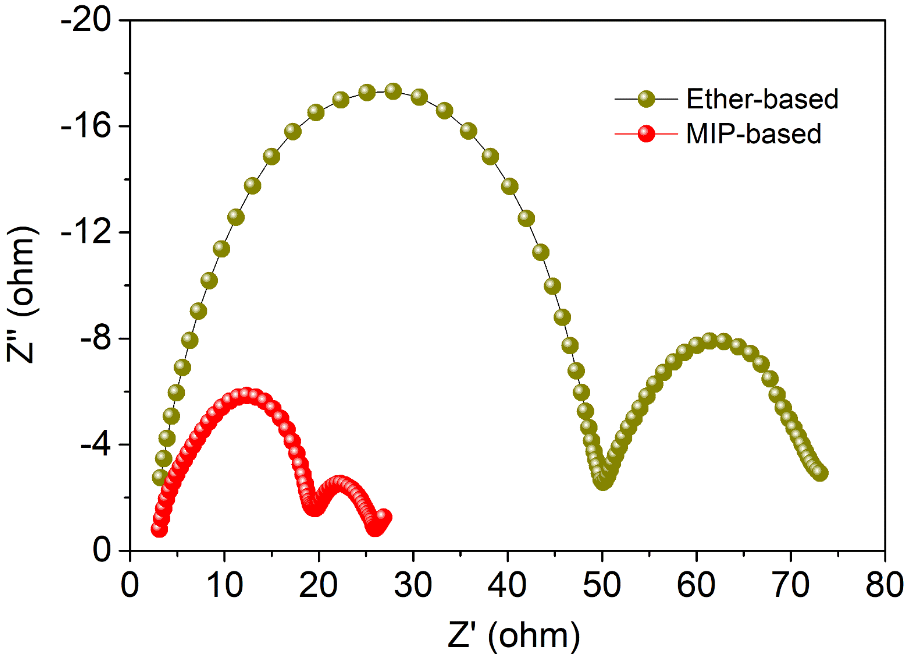

Supplementary Figure 12 |**Z-K spectra for symmetrical Li||Cu after initial plating lithium deposition.**

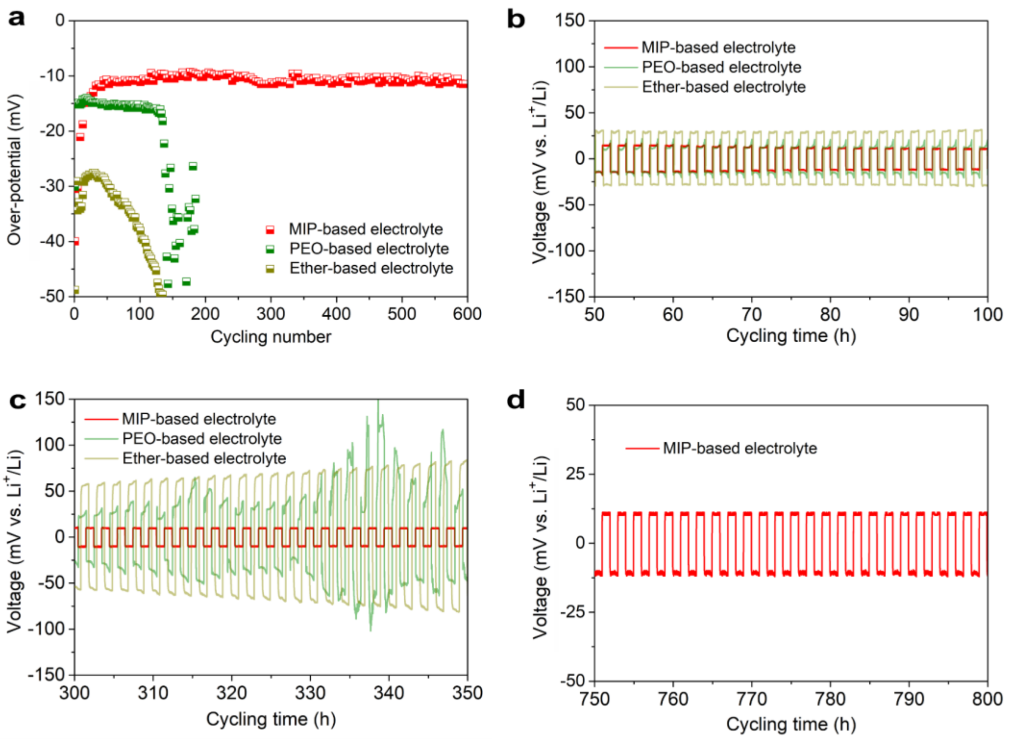

Supplementary Figure 13 | **The electrochemical performance of symmetrical Li||Li cells. a** the over-potential and **b-d** the corresponding enlargements for different cycling time from Fig. 3g. The results show that the MIP-based electrolyte trends to deliver a stable over-potential (~ 10 mV) even after 600 cycles (1200 h), however, obvious voltage fluctuation can be found in other electrolyte system. For instance, the over potential of the cell using ether-based electrolyte can reach up to ~ 40 mV, in contrast, the over potential of MIP-based electrolyte still remains at ~ 10 mV after 100 cycles (200 h).

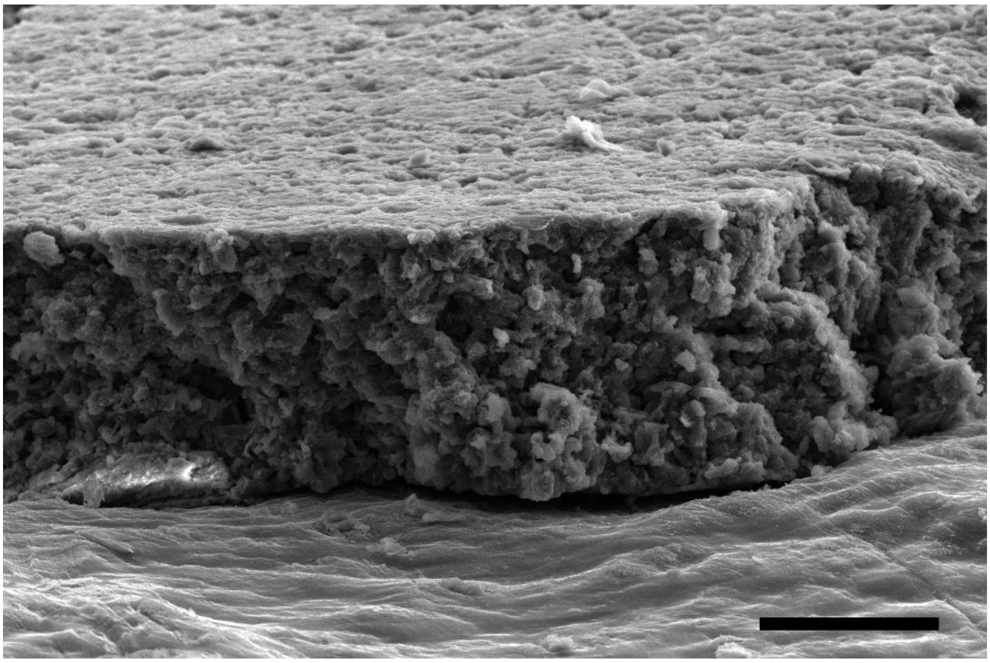

Supplementary Figure 14 | **The cross-sectional for Li||Li cells with MIP-based electrolyte after 1200 h cycling.** Scale bar, 25 μm.

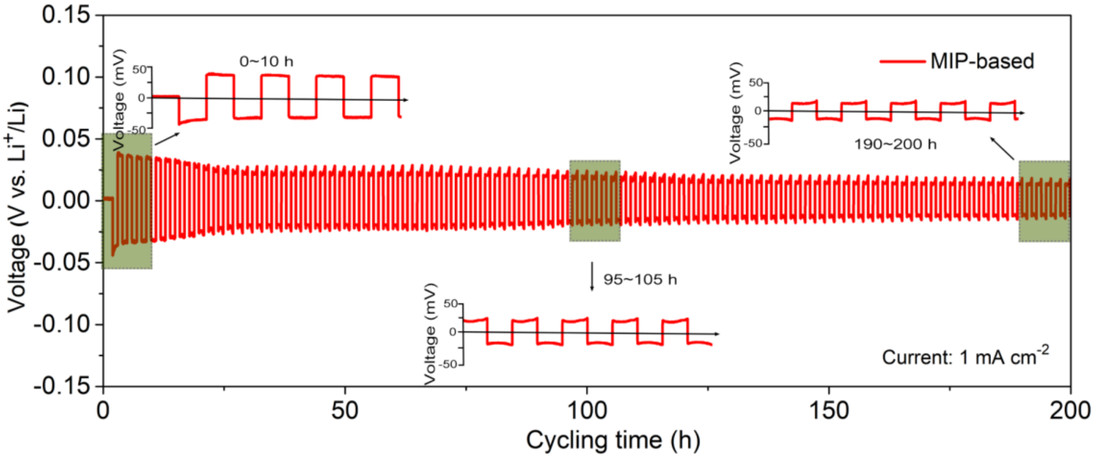

Supplementary Figure 15 | **Cycling performance of symmetrical Li||Li cell.** The electrolyte is based on MIP-based electrolyte after resting for one month in glove box. The results show that the symmetrical Li||Li cells still deliver stable cycle in the MIP-based electrolyte for 200 h (tests were stopped at that point) at 1.0 mA cm^{-2} even after resting for one month, confirming the stability of the montmorillonite dispersion in the electrolyte.

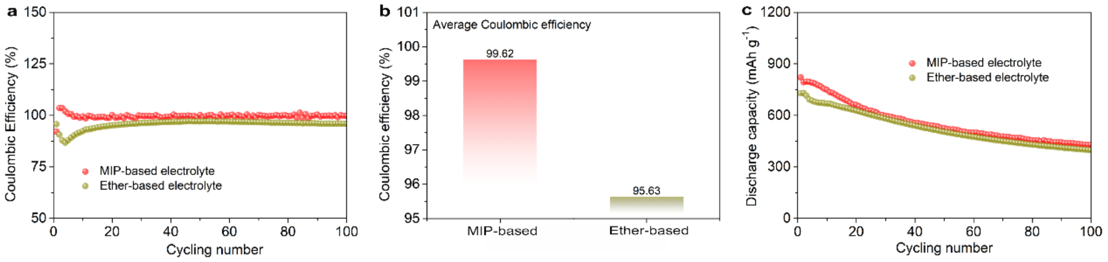

Supplementary Figure 16 | **Electrochemical performace of lithium-sulfur battery. a** The Coulombic efficiency, **b** the average Coulombic efficiency and **c** the cycling performance based on MIP- and ether-based electrolye. The current density for testing is 0.5 mA cm^{-2}. These results clearly demonstrate the role of MIP-based electrolyte in stabilizing Li metal to achieve higher average Coulombic efficiency (99.62 %) than of the cell using ether-based electrolyte (95.63 %) during 100 cycles.

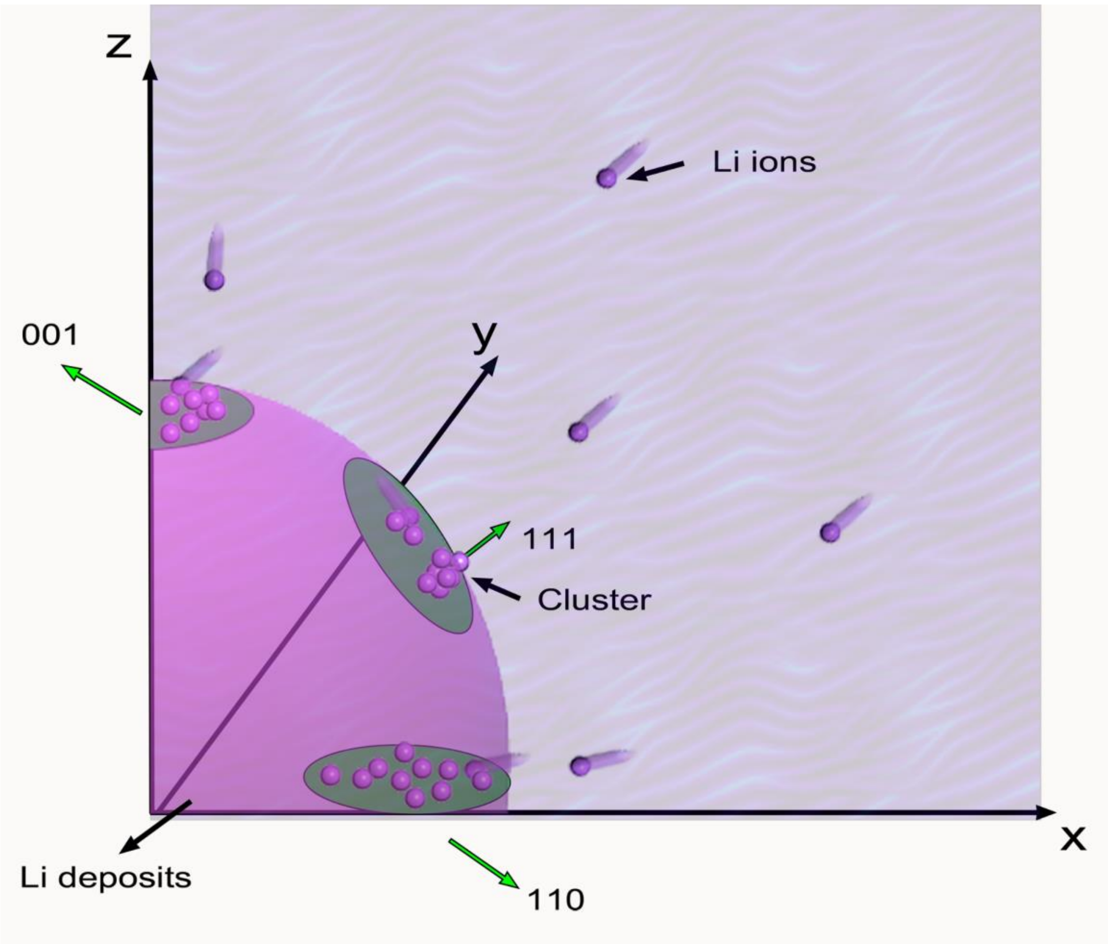

Supplementary Figure 17 | **Schematic illustration describing Li ion plating behavior on different crystal planes**. The Li ions prefer stack and growth on the Li <111> crystal plane to form clusters or dendrites due to the higher diffusion barrier than that on the other crystal plane.

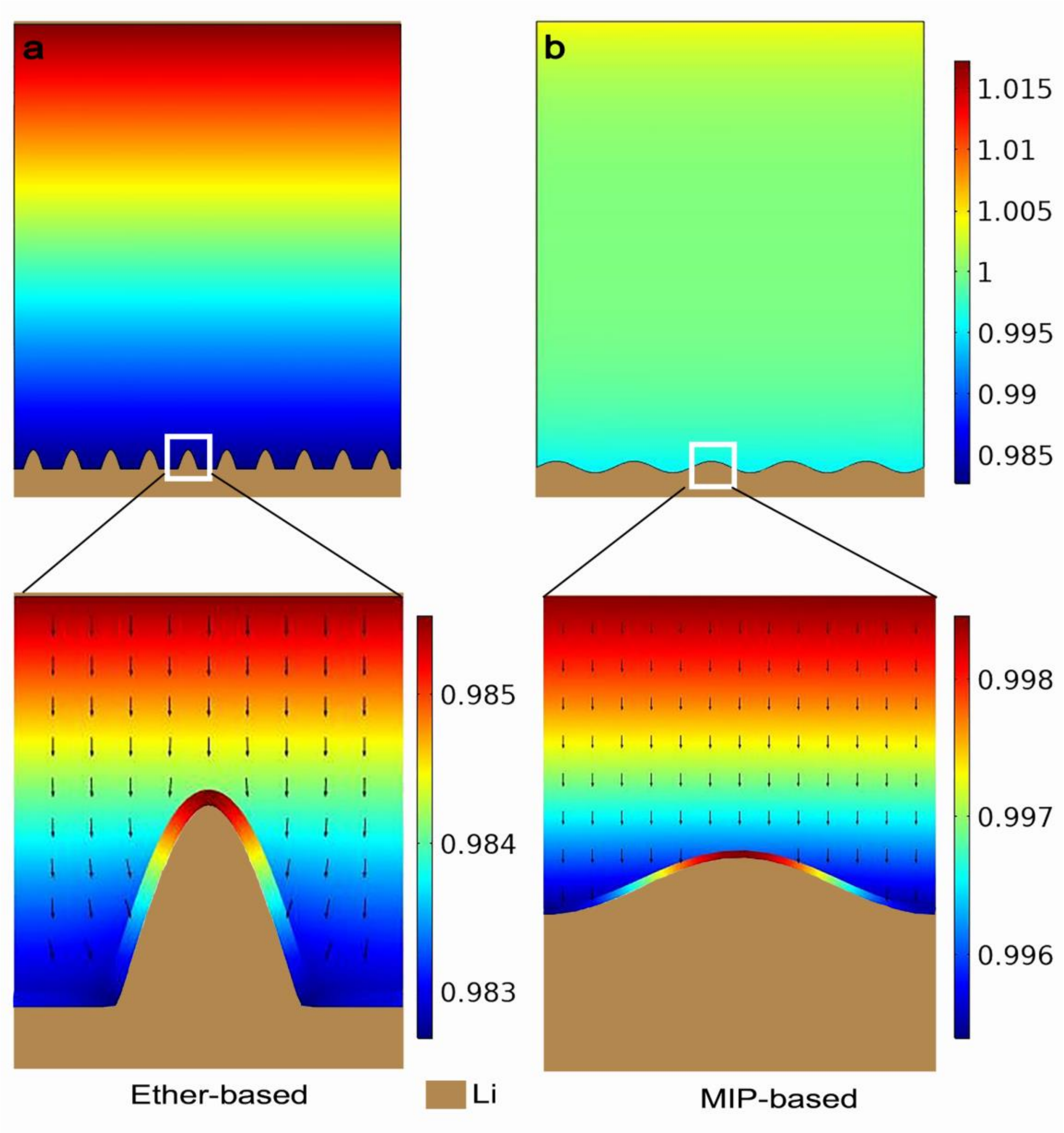

Supplementary Figure 18 | **Equilibrium Li-ion concentration profiles. a** The ether-based and **b** the MIP-based electrolyte at current density of 0.5 mA cm^{-2}. The simulated morphology is based on the experimentally observed morphology in Figs. 2d, h. The scale of constant-reaction-current electrode surfaces is 20 × 25 μm^2.

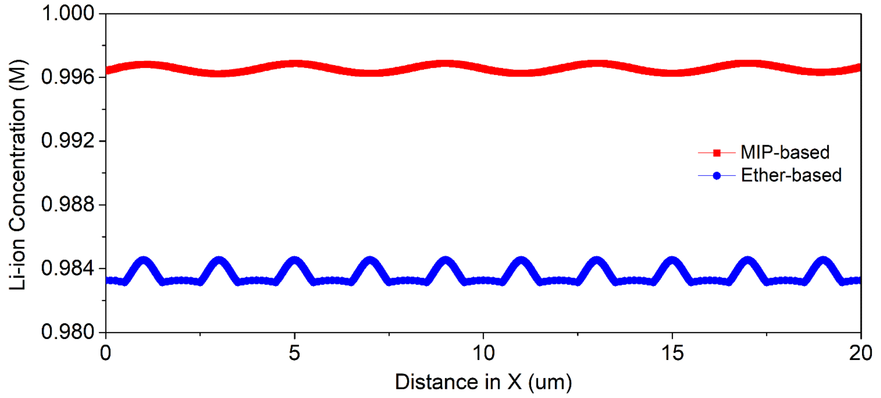

Supplementary Figure 19 | **Li-ion concentration along the X direction in supplementary Fig. 18.**

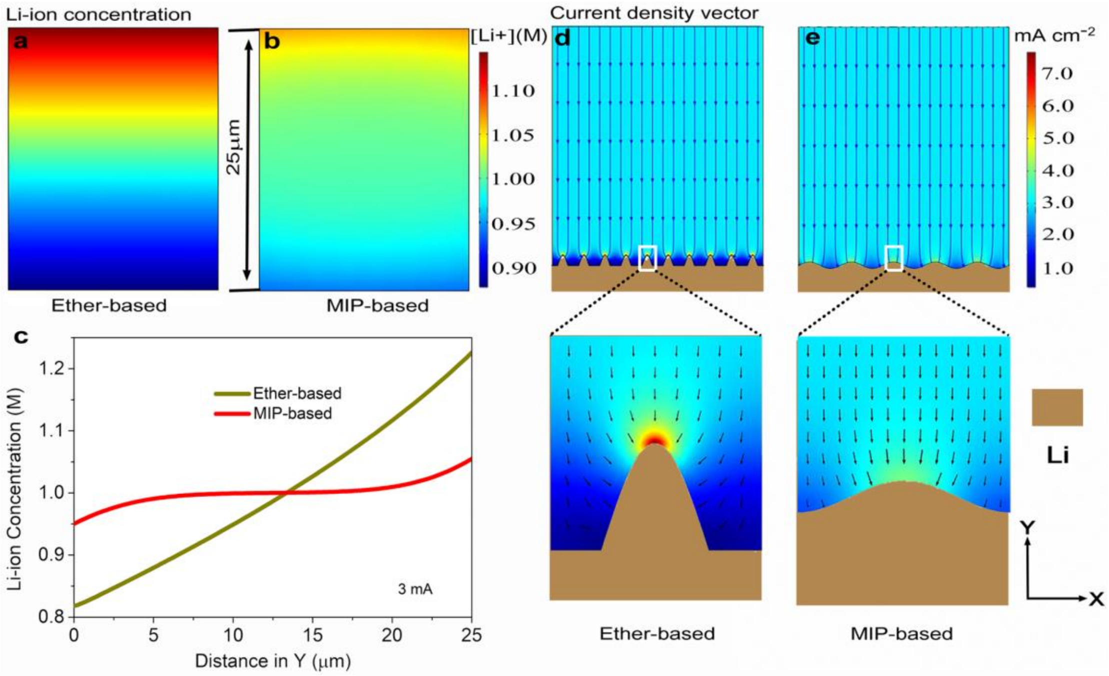

Supplementary Figure 20 | **Equilibrium Li-ion concentration profiles. a** The ether- and **b** the MIP-based electrolyte at a high current density of 3 mA cm^{-2}. The results further demonstrate the ability of MIP-based electrolyte to allow the homogeneous distribution of lithium ion at the surface of anode even at a high current density of 3 mA cm^{-2}. The scale of constant-reaction-current electrode surface is $20 \times 25\ \mu m^2$.

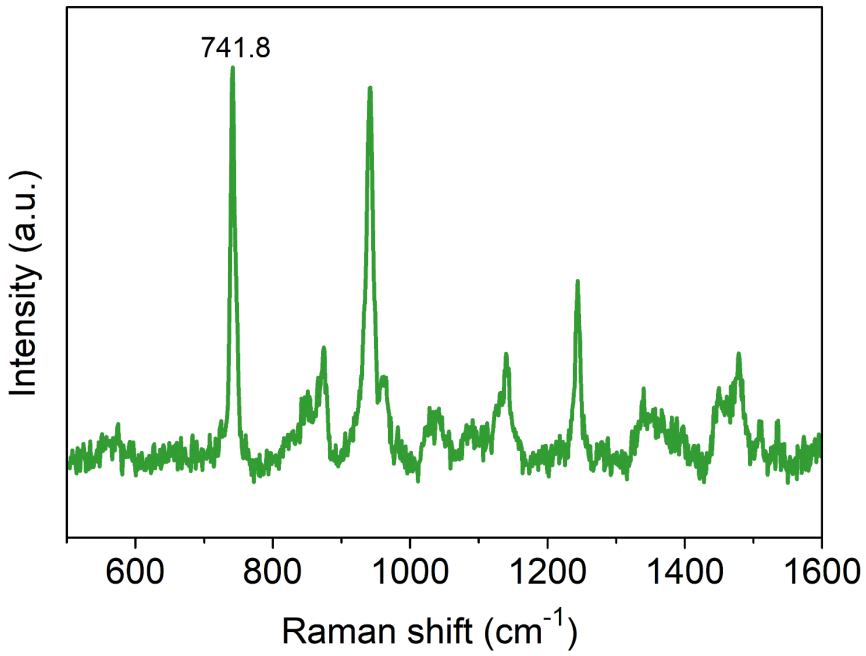

Supplementary Figure 21 | **The complete Raman spectrum for ether-based electrolyte.** The Raman shift at 741.8 cm^{-1} originates from $TFSI^{-}$ bond.

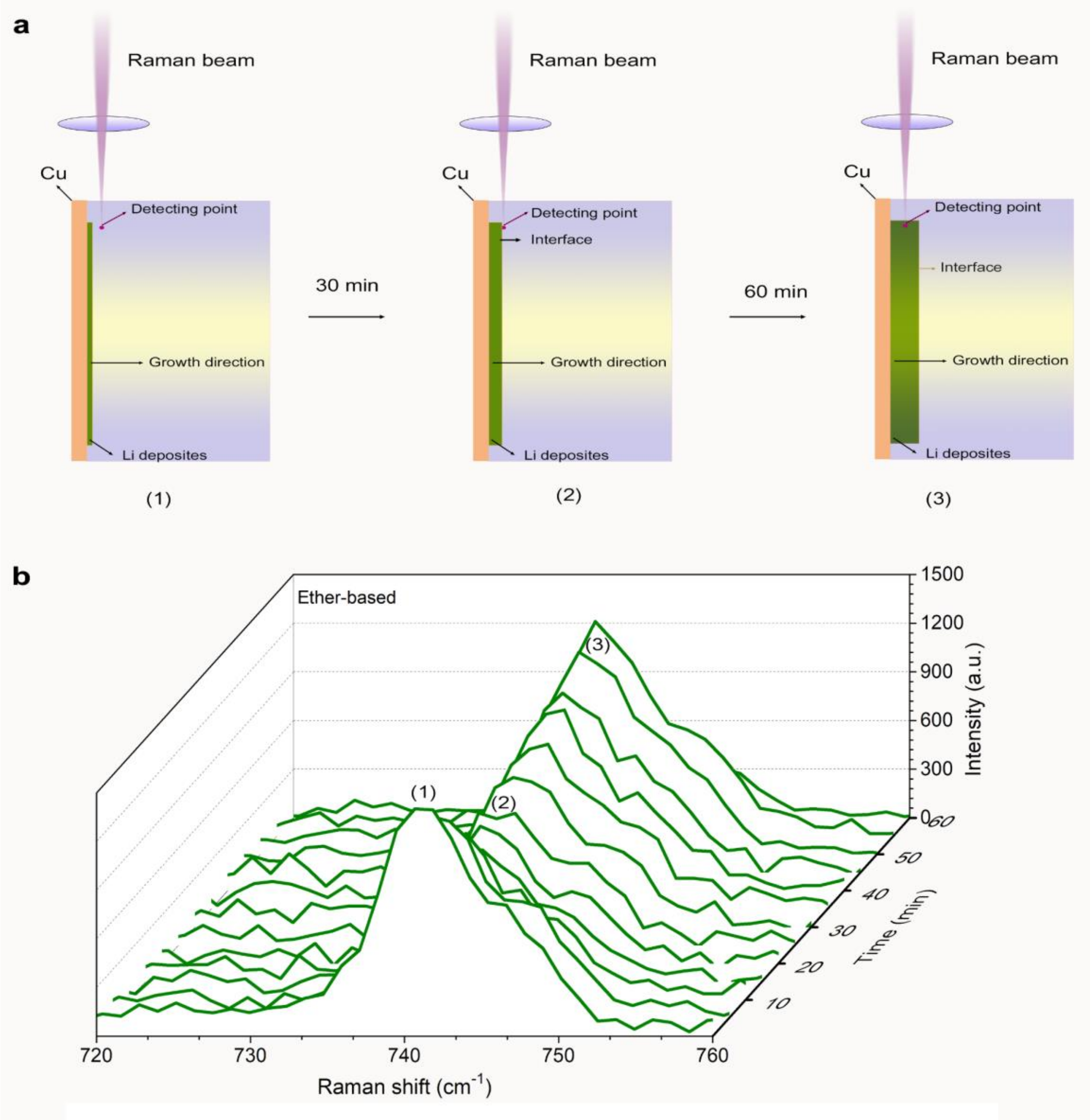

Supplementary Figure 22 | **Schematic diagram of *in-situ* Raman measurement**. **a** The Raman beam is foucsed on the neiborhood of Cu cross section, resulting in the initial measered results are from the bulk electrolyte (marked (1)), and then, during the Li platting, the interface of deposits/electrolyte gradully moves along the lithium deposits direction and then arrive at the foucsed point of Raman beam (marked (2)), where Raman signal for the reliable interface between solid and liquid can be obtain to imply the ion destribution[1]. At last, once the reliable interface keep away the Raman foucsed point, the detecting signal for bulk electrolyte can be restore (marked (3)). **b** *In-situ* Raman spectrum of ether-based electrolyte for $TFSI^-$ bond at 741.8 cm^{-2}. The intensity shows fast decay after 30 min plating,

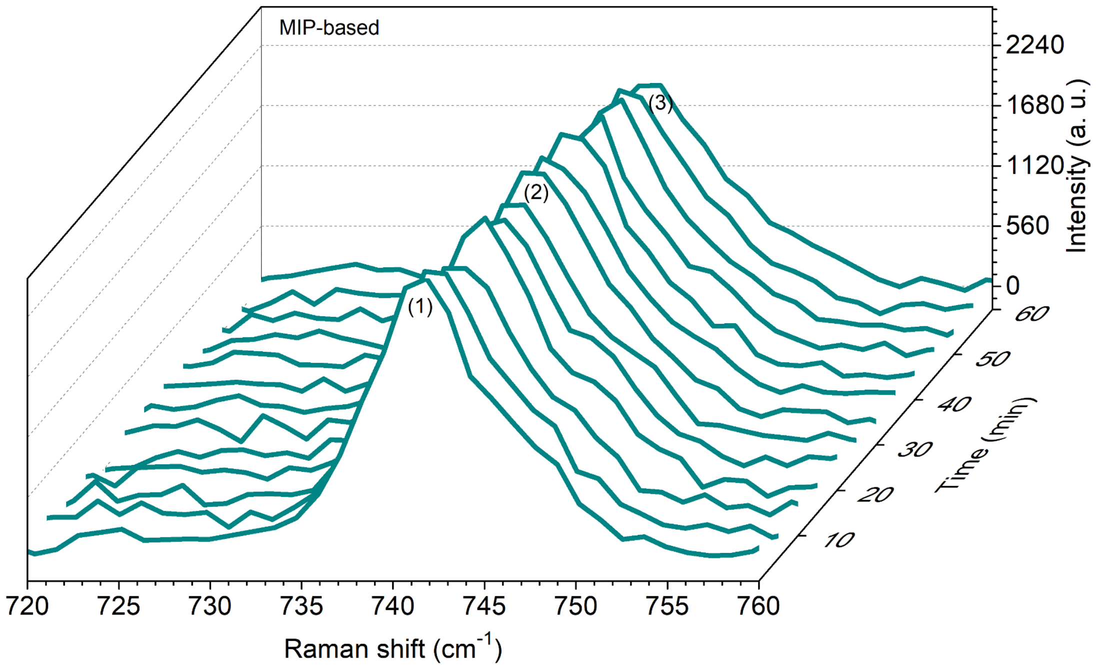

Supplementary Figure 23 | ***In-situ* Raman spectrum of MIP-based electrolyte for TFSI⁻ bond at 741.8 cm^{-1}.** No decay of the intensity can be found during cycling, implying stable ion destribution without any influence by electric filed in MIP-based electroylte during plaiting.

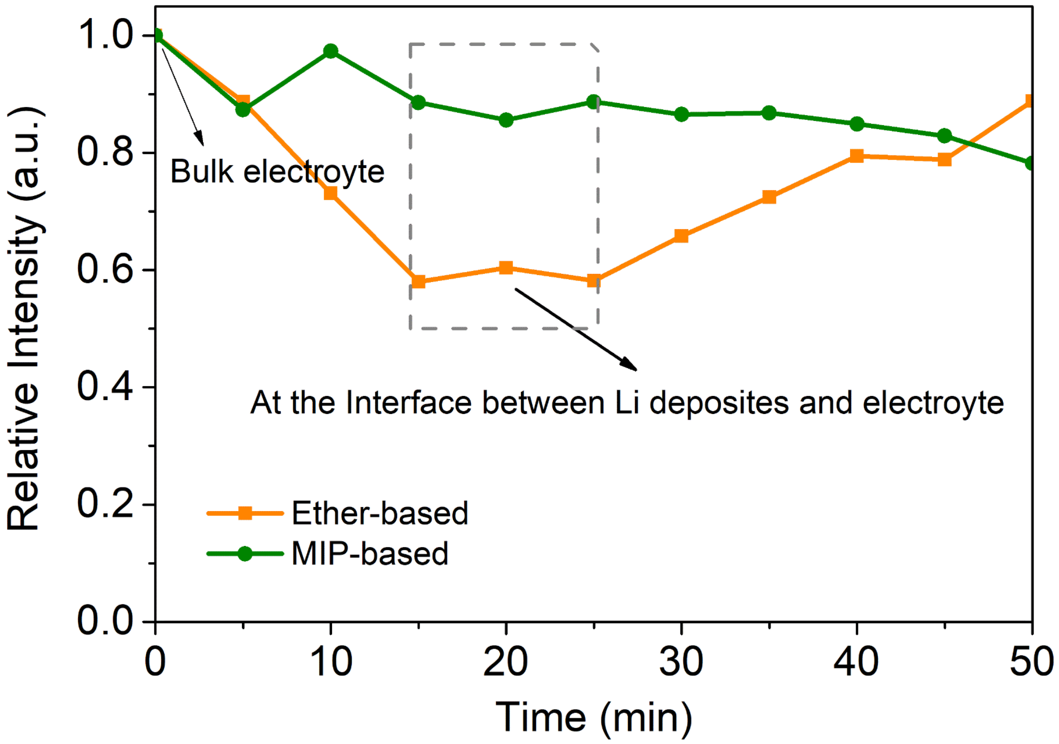

Supplementary Figure 24 | **The comparsion of relative intensity of TFSI$^-$ Raman bond.**

Supplementary Reference

1. Lei, T. et al. Inhibiting polysulfide shuttling with a graphene composite separator for highly robust lithium-sulfur batteries. *Joule* **2**, 2091–2104 (2018).
